# Chlamydial Protease-Like Activity Factor and Type III Secreted Effectors Cooperate in Inhibition of p65 Nuclear Translocation

**DOI:** 10.1128/mBio.01427-16

**Published:** 2016-09-27

**Authors:** Michael John Patton, Stuart McCorrister, Chris Grant, Garrett Westmacott, Robert Fariss, Pingzhao Hu, Kaiqiong Zhao, Mary Blake, Bill Whitmire, Chunfu Yang, Harlan D. Caldwell, Grant McClarty

**Affiliations:** a Laboratory of Clinical Infectious Diseases, National Institutes of Health, Bethesda, Maryland, USA; b National Microbiology Laboratories, Winnipeg, Manitoba, Canada; c National Eye Institute, National Institutes of Health, Bethesda, Maryland, USA; d Department of Biochemistry and Medical Genetics, University of Manitoba, Winnipeg, Canada; e National Institute of Arthritis and Musculoskeletal and Skin Diseases, National Institutes of Health, Bethesda, Maryland, USA

## Abstract

The chlamydial protease-like activity factor (CPAF) is hypothesized to be an important secreted virulence factor; however, challenges in denaturing its proteolytic activity have hampered attempts to identify its legitimate targets. Here, we use a genetic and proteomic approach to identify authentic CPAF targets. Human epithelial cells infected with CPAF-sufficient and CPAF-deficient chlamydiae were lysed using known CPAF-denaturing conditions. Their protein profiles were analyzed using isobaric mass tags and liquid chromatography-tandem mass spectrometry. Comparative analysis of CPAF-sufficient and CPAF-deficient infections identified a limited number of CPAF host and chlamydial protein targets. Host targets were primarily interferon-stimulated gene products, whereas chlamydial targets were type III secreted proteins. We provide evidence supporting a cooperative role for CPAF and type III secreted effectors in blocking NF-κB p65 nuclear translocation, resulting in decreased beta interferon and proinflammatory cytokine synthesis. Genetic complementation of null organisms with CPAF restored p65 nuclear translocation inhibition and proteolysis of chlamydial type III secreted effector proteins (T3SEs). We propose that CPAF and T3SEs cooperate in the inhibition of host innate immunity.

## IMPORTANCE

*Chlamydia trachomatis* is an important human pathogen responsible for over 100 million infections each year worldwide. Its success as an intracellular pathogen revolves around its ability to evade host immunity. The chlamydial protease-like activity factor (CPAF) is a conserved serine protease secreted into the host cytosol of infected cells that is thought to play an important role in immune evasion. Currently, CPAF’s authentic *in situ* target(s) and mechanism of action in immune evasion are poorly characterized. Using a CPAF-deficient strain and high-throughput proteomics, we report novel CPAF host and chlamydial targets. Host targets were primarily interferon-stimulated genes, whereas chlamydial targets were exclusively type III secreted proteins. We propose a novel mechanism for CPAF and type III secreted proteins in the evasion of host innate immune responses. These findings provide new insights into CPAF’s function as a virulence factor and a better understanding of how chlamydiae evade host immunity.

## INTRODUCTION

*Chlamydia trachomatis* causes over 100 million new infections each year worldwide (1). It is the major cause of bacterial sexually transmitted infections (STIs) and trachoma, the leading cause of preventable infectious blindness (1). Despite its global medical importance, there is no licensed chlamydial vaccine. A vaccine has been elusive due to poor natural protective immunity following infection (2). Development of a vaccine requires a better understanding of chlamydial virulence factors that suppress host defenses.

*C. trachomatis* is an obligate intracellular bacterium characterized by a unique biphasic developmental life cycle (3). The elementary body (EB) initiates infection by entering host cells by phagocytosis and resides in a protective vacuolar niche termed an inclusion. Within the inclusion, the EB differentiates into a noninfectious, metabolically active reticulate body (RB). The RB replicates by binary fission, generating numerous progeny that undergo a secondary differentiation back to infectious EBs. Following lysis of host cells, EBs are released and repeat the infectious life cycle.

The type III secretion system (T3SS), termed an injectisome, is shared among many Gram-negative pathogens (4). Its primary function is to secrete type III secreted effector proteins (T3SEs) into the host cytosol, which modulate cell functions to ensure pathogen survival (4). Chlamydiae possess two functionally distinct categories of T3SEs termed early (0- to 12-h) and midcycle (12- to 36-h) effectors (5). EBs secrete preloaded early effectors that function in cell invasion, immune evasion, and inclusion membrane (IM) biogenesis (6). Populations of RBs closely positioned at the inclusion membrane secrete midcycle effectors into the host cell cytosol from a structurally unique injectisome that spans the RB outer membrane and the IM (6). Activation of the RB midcycle injectisome and the function of midcycle T3SEs are poorly characterized (7).

Midcycle RBs produce chlamydial protease-like activity factor (CPAF) as an inactive zymogen (CPAFi). Following type II secretion (T2S), CPAF autocatalyzes into an active protease (CPAFa) (8–11). CPAF has been the focus of investigation because it is secreted and conserved among chlamydiae. Prior reports implicated CPAF in the proteolysis of innate transcription factor NF-κB subunit p65 (12) and adaptive transcription factors USF-1 and RFX5 (13) and maintenance of pathogen vacuole integrity (14). Due to the challenges of inactivating CPAF’s potent proteolytic activity prior to experimentation, these targets have now been shown to be nonauthentic substrates (15). Thus, despite the consensus that CPAF is a critical virulence factor, the protease’s authentic *in situ* target(s) remains poorly defined.

Here, we conducted a comparative proteomic study of human cervical epithelial cells infected with a *C. trachomatis* CPAF-deficient strain, RST-17 (here referred to as null), and a CPAF-sufficient strain, RST-5 (here referred to as wild type [WT]) to identify legitimate chlamydial and host CPAF targets (8). Using a combined proteomic and genetic approach, we report that CPAF targets a restricted number of both chlamydial and host proteins. Host targets were predominantly interferon-stimulated gene products (ISGs) and chlamydial targets T3SEs. We show that CPAF and T3SEs function cooperatively in suppressing innate immune signaling by inhibiting p65 nuclear translocation. CPAF complementation of the null strain (referred to as L2-17/CPAF) restored p65 nuclear translocation inhibition and proteolysis of chlamydial T3SEs, confirming a cooperative role for CPAF and T3S proteins in the inhibition of the host innate immune response. As a result, we propose a model for a cooperative interaction between CPAF and chlamydial T3S in the inhibition of p65 nuclear translocation.

## RESULTS

### Comparative proteomics of WT- and CPAF null-infected cells identifies T3S proteins as CPAF targets.

**FIG 1:**
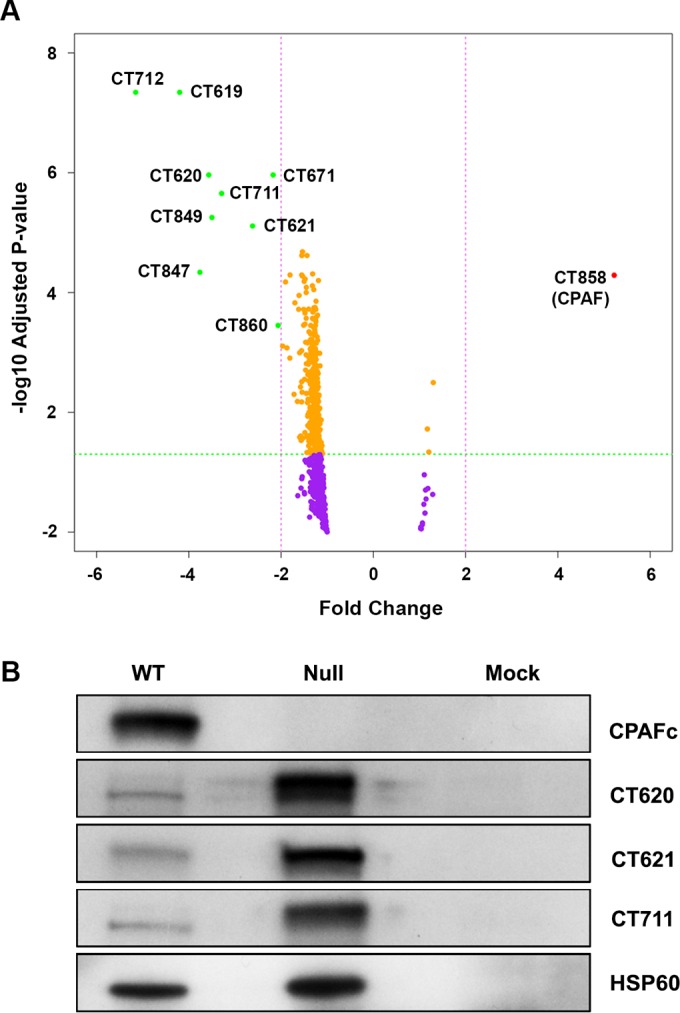
CPAF effects T3S proteins. (A) Volcano plot proteomic comparison of WT with null infections shows 675 chlamydial proteins. The 665 proteins represented as yellow and purple dots did not meet the threshold of either a >2-fold change in TMT intensity (pink dashed line) or an adjusted *P* value of <0.05 (green dashed line). Nine green-labeled proteins with a >2-fold decrease in TMT intensity are exclusively T3SEs or T3SS proteins. The only protein found with a >2-fold change in TMT intensity between the WT and null infections was CT858 (CPAF), labeled in red. (B) Western blot analysis of T3SE CT620, CT621, CT711, and CPAFc confirms chlamydial proteins exhibiting a >2-fold difference in TMT intensity between WT and null infections. Chlamydial HSP60 served as a loading control.

Protein lysates from WT-, null-, and mock-infected HeLa 229 cells (HeLa) were equivalently labeled with tandem mass tags (TMTs) and analyzed by nanoflow liquid chromatography-tandem mass spectrometry (nLC-MS/MS). A total of 675 chlamydial proteins were identified (Fig. 1A; see also Table S1 in the supplemental material). Remarkably, employing a >2-fold change in mean TMT intensity and an adjusted *P* value of <0.05 across four replicates with 99% confidence of 2-peptide identification, only 10 chlamydial proteins (CT619, CT620, CT621, CT671, CT711, CT712, CT847, CT849, CT858, and CT860) were found to differ between WT and null infections (Fig. 1A). Seven of the 10 chlamydial proteins identified in lower abundance in WT infections were midcycle T3SEs containing domains of unknown function (DUF): CT619, CT620, CT621, CT711, CT712, CT847, and CT849. While T3SE host targets remain largely unknown, CT620, CT621, and CT711 have been shown to be secreted midcycle into the host cytosol and localized in the host nucleus (7). As previously described, CT620 and CT711 display two polypeptide bands by Western blotting: an unstable higher-molecular-weight form and a more stable lower-molecular-weight form (7). Notably, the higher-molecular-weight polypeptide is much more abundant in null infections than in WT infection (Fig. 1B). CT847, a known T3SE, was found in lower abundance in WT infections (6). In chlamydiae, the site of interaction between the RB T3S needle and the IM is termed the translocation pore (16). CT860 is a part of a family of T3S proteins thought to function at the IM translocation pore and was found to be in lower abundance in WT infections (16). CT671, a structural homologue to the T3S needle length regulator YscP of *Yersinia pestis*, was also found to be in lower abundance in the WT infections. CT858 (CPAF) was the only chlamydial protein with a >2-fold abundance change (5.22-fold) when comparing WT and null infections. Intriguingly, above the 1.5-fold threshold and the adjusted *P* value of <0.05, T3SS class III chaperones (CT665 and CT667) and a translocation chaperone (CT579) were less abundant in WT infections (see Table S2 in the supplemental material). Western blotting verified proteomic T3SE hits CT620, CT621, and CT711 (Fig. 1B). WT infections expressing CPAFa were confirmed using a C-terminal 35-kDa CPAF antibody (referred to as CPAFc [Fig. 1B]) (17).

### Comparative proteomics of WT and CPAF null infections identifies host proteins functional in innate immunity as CPAF targets.

**FIG 2:**
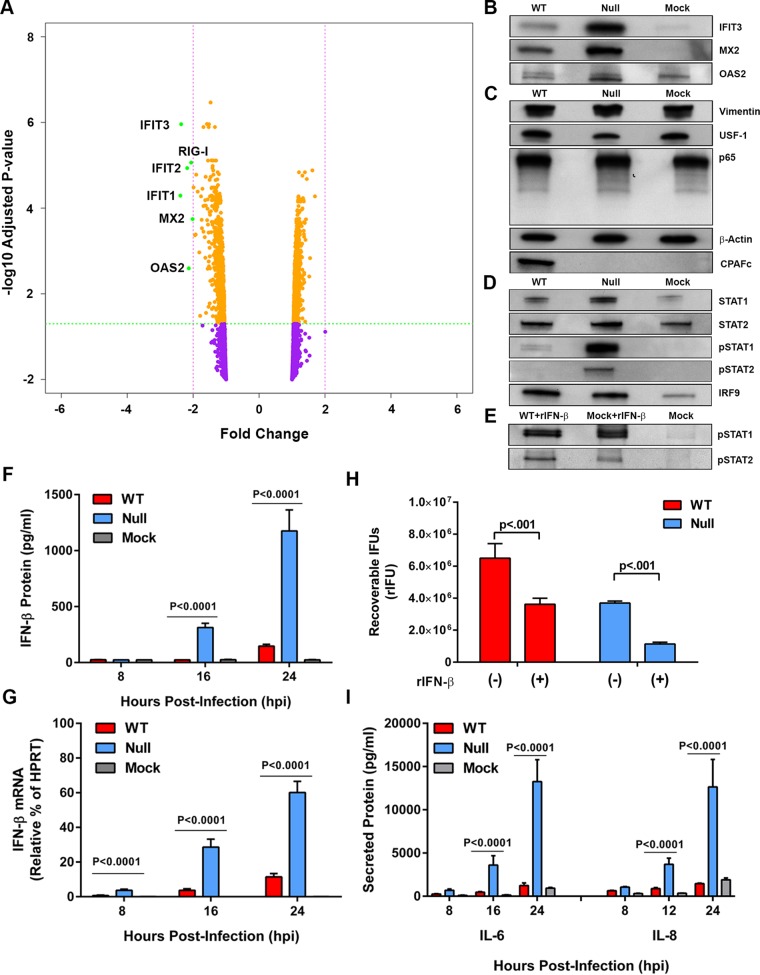
Host proteome, type I interferon, and proinflammatory signaling in WT- and null-infected cells. (A) Volcano plot proteomic comparison of WT with null infections shows 6,228 host proteins. The 6,222 proteins represented as yellow and purple dots did not meet the threshold of either a >2-fold change in TMT intensity (pink dashed line) or an adjusted *P* value of <0.05 (green dashed line). Six green-labeled proteins with a >2-fold decrease in TMT intensity are ISGs and a PRR. No host proteins were found with a >2-fold increase in TMT intensity between the WT and null infections. (B) Western blot analysis of ISGs IFIT3, MX2, and OAS2 confirms host proteins exhibiting a >2-fold decrease in TMT intensity between WT and null infections. (C) Western blot analysis of previously identified nonauthentic targets of CPAF (vimentin, USF-1, and p65) shows no proteolytic degradation, thus confirming CPAF inactivation. β-Actin served as a loading control. CPAFc is visible in WT and not null or mock infections. (D) Western blot analysis of JAK-STAT signaling shows tyrosine phosphorylation of STAT1 and STAT2 in the null- but not WT-infected cells. (E) WT-infected cells treated with rIFN-β show tyrosine phosphorylation of STAT1 and STAT2. (F) IFN-β in cell culture supernatants of WT-, null-, and mock-infected cells at different hours postinfection. Null-infected cells produce significantly more IFN-β than WT-infected cells at 16 and 24 hpi. Statistical significance was determined across four replicates via a 2-way analysis of variance. (G) IFN-β mRNA in WT-, null-, and mock-infected cells at different hours postinfection. Null-infected cells produce significantly more IFN-β mRNA than WT at 16 and 24 hpi. Statistical significance was determined across four replicates via a 2-way analysis of variance. HPRT, hypoxanthine phosphoribosyltransferase. (H) rIFU of WT- and null-infected cells treated and not treated with rIFN-β. Both WT and null organisms are sensitive to pretreatment. Null-infected cells were more sensitive to pretreatment, consistent with the observation that they produce more IFN-β (F). Statistical significance was determined across three replicates by multiple *t* test analysis. (I) Proinflammatory cytokines IL-6 and IL-8 in the supernatants of WT-, null-, and mock-infected cells at different hours postinfection. Null-infected cells produce significantly more IL-6 and IL-8 than do WT-infected cells. Statistical significance was determined across four replicates via a 2-way analysis of variance. All analyses of variance and *t* test analyses were performed using GraphPad Prism 6.

The proteomic analysis of WT-, null-, and mock-infected cells identified a total of 6,228 host proteins, of which only six proteins had a >2-fold change in TMT intensity and an adjusted *P* value of <0.05 in WT versus null infections (Fig. 2A). The six proteins identified were cytosolic pattern recognition receptor (PRR) retinoic acid-inducible gene I (RIG-I/DDX58) and ISGs (IFIT1 to -3, MX2, and OAS2). IFIT3, MX2, and OAS2 were analyzed by Western blotting to verify the proteome findings. Western blot assays showed a lower abundance of IFIT3, MX2, and OAS2 in WT than in null infections (Fig. 2B). Importantly, we observed no detectable cleavage products of the host proteins vimentin, USF-1, and NF-κB subunit p65 (Fig. 2C), validating the inactivation of CPAF’s nonspecific proteolytic activity following hot SDS lysis of infected cells. Thus, similar to the chlamydial proteome, a very restricted number of host protein targets was found to differ between WT and null infections. Based on these results, we hypothesized that WT and null organisms differed in their ability to induce type I interferons (IFN-I) and proinflammatory cytokines. To test this hypothesis, lysates of WT-, null-, and mock-infected cells were analyzed by Western blotting using antibodies specific to proteins of the Janus kinase-signal transducer and activator of transcription (JAK-STAT) pathway. The results show that both WT- and null-infected cells expressed equivalent amounts of STAT2 and IRF9. We observed a reduction in STAT1 in WT-infected cells. Interestingly, there was a significant reduction in tyrosine phosphorylation of STAT1 (pSTAT1) and STAT2 (pSTAT2) in WT-infected cells (Fig. 2D); however, WT-infected cells retained the ability to phosphorylate STAT1 and STAT2 following treatment with 100 U recombinant human beta interferon (rIFN-β) (Fig. 2E). This result showed that the JAK-STAT signaling pathway was functional in WT-infected cells and that the lack of STAT1 and STAT2 phosphorylation was likely the result of reduced IFN-β expression. WT-infected cells produced >10-fold less IFN-β (Fig. 2F) and 60% less IFN-β mRNA than null-infected cells (Fig. 2G). Treatments of WT- and null-infected cells with 100 U of rIFN-β had similar effects on growth, with both strains showing a 50% reduction in recoverable infection-forming units (rIFU) (Fig. 2H). We similarly found that WT-infected cells secreted >10-fold less interleukin 6 and 8 (IL-6 and IL-8, respectively) than did null-infected cells (Fig. 2I). These results clearly establish a role for CPAF in the inhibition of IFN-I and proinflammatory cytokine synthesis.

### Nuclear translocation of NF-κB subunit p65 is inhibited in WT-infected cells.

**FIG 3:**
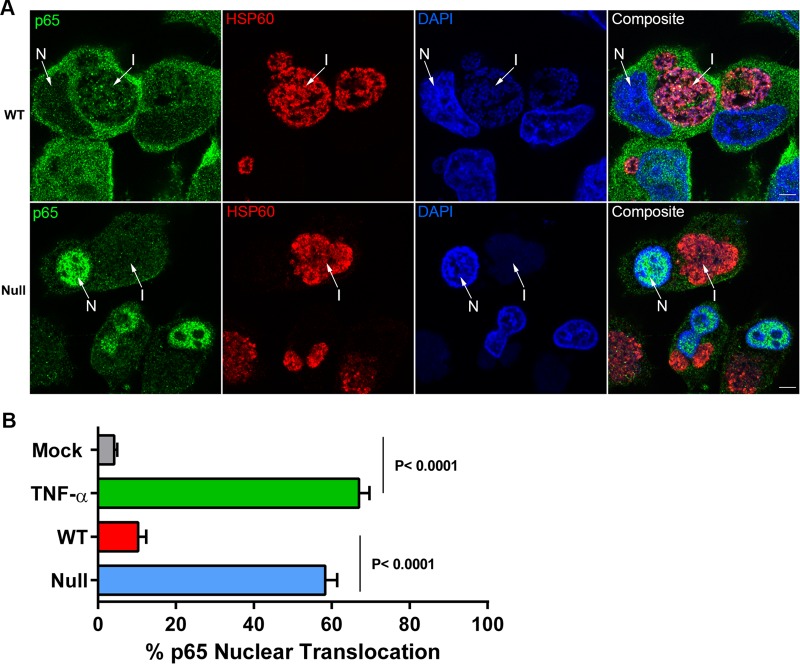
WT organisms block p65 nuclear translocation. (A) Airyscan high-resolution imaging of WT- and null-infected HeLa cells stained with anti-p65, anti-HSP60, and DAPI. p65 staining, shown in green, was localized in the cytoplasm around the nucleus (N) and inclusion (I) in WT-infected cells and intensely localized to the host nuclei (N) in null-infected cells (arrows). HSP60 staining, shown in red, was localized to chlamydial inclusions (I) in both WT- and null-infected cells. DAPI staining, shown in blue, depicts host nuclei (N) and chlamydial inclusions (I) of WT- and null-infected cells. Composite images are shown in the final panel. Bars, 5 µm. (B) p65 nuclear translocation in mock, TNF-α-treated, WT, and null infections counted in quadruplicate replicates (*n* = 200 cells per replicate). TNF-α treatment results in >55% p65 nuclear translocation. A similar percentage was found in null-infected cells. In contrast, <15% of WT-infected cells exhibited p65 nuclear translocation. Statistical significance was determined across four replicates via a 1-way analysis of variance. Analysis of variance was performed using GraphPad Prism 6.

A logical target for CPAF-mediated T3SE(s) inhibition of IFN-I and proinflammatory cytokine synthesis is the transcriptional factor NF-κB subunit p65. We pursued this potential mechanism using confocal microscopy to exclude nonspecific CPAF effects on p65 nuclear translocation which could occur during nuclear isolation. Confocal images of p65 nuclear translocation in WT- and null-infected cells are shown in Fig. 3A. Infected cells were stained with anti-p65, chlamydial anti-HSP60, and 4′,6-diamidino-2-phenylindole (DAPI). p65 staining in WT-infected cells was primarily located in the cytoplasm. There was minimal nuclear p65 staining. In contrast to these results, p65 staining in null-infected cells was mostly nuclear in nature. The percentage of p65 nuclear translocation in null-infected and tumor necrosis factor alpha (TNF-α)-treated cells was >55%, whereas <15% of the cells exhibited p65 nuclear translocation in WT-infected cells (Fig. 3B).

### CPAF complementation of null mutant strain restores p65 nuclear translocation inhibition.

**FIG 4:**
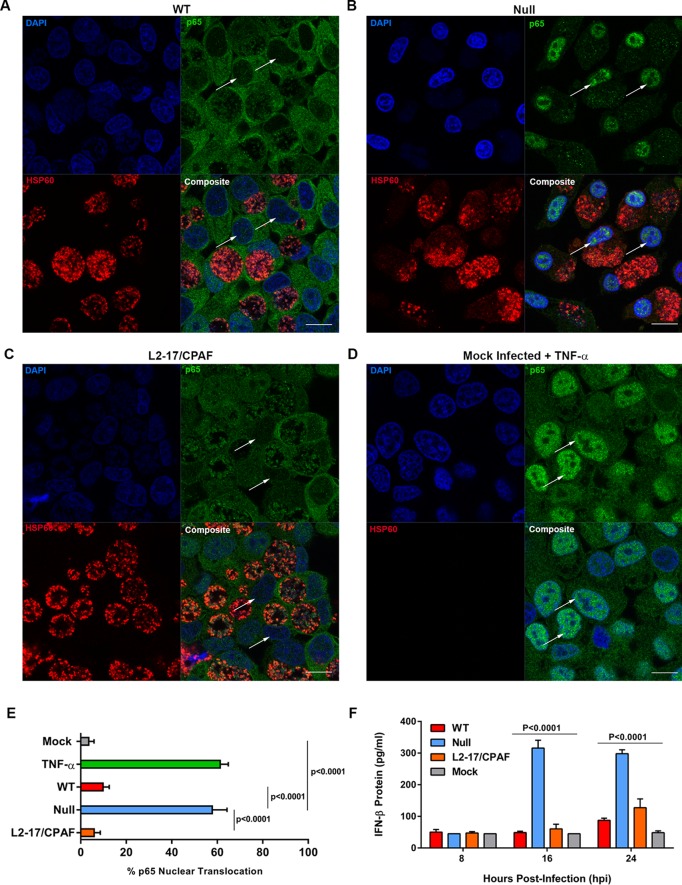
CPAF complementation restores p65 nuclear translocation inhibition. (A to D) Airyscan high-resolution imaging of WT-, null-, and L2-17/CPAF complemented organism-infected HeLa cells stained with anti-p65, anti-HSP60, and DAPI. p65 staining, shown in green, was localized in the cytoplasm around the nucleus and inclusion in WT- and L2-17/CPAF-infected cells and intensely localized to the host nuclei in null-infected cells (arrows designate nuclei). HSP60 staining, shown in red, was localized to chlamydial inclusions in all infections. DAPI staining, shown in blue, depicts host nuclei and chlamydial inclusions of all infections. Composite images are shown in each respective infection. HeLa cells treated with 150 ng of TNF-α/ml serve as a positive control for nuclear translocation of p65 (arrows designate nuclei). Bars, 20 µm. (E) p65 nuclear translocation in mock, TNF-α treatment, WT, null, and L2-17/CPAF infections counted in quadruplicate replicates (*n* = 200 cells per replicate). TNF-α treatment results in >60% p65 nuclear translocation. A similar percentage was found in null-infected cells. In contrast, <15% of WT- and L2-17/CPAF-infected cells exhibited p65 nuclear translocation. Statistical significance was determined across four replicates via a 1-way analysis of variance. Analysis of variance was performed using GraphPad Prism 6. (F) IFN-β in cell culture supernatants of WT-, null-, L2-17/CPAF-, and mock-infected cells at different hours postinfection. Null-infected cells produce more IFN-β than WT- and L2-17/CPAF-infected cells at 16 and 24 hpi. Statistical significance was determined across four replicates via a 2-way analysis of variance.

In order to confirm the host and chlamydial phenotypes associated with the CPAF gene, the null strain was complemented with CPAF expressed on the recombinant chlamydial plasmid (L2-17/CPAF) (18). HeLa cells grown on coverslips infected with WT, null, and L2-17/CPAF organisms were probed with anti-p65, chlamydial anti-HSP60, and DAPI. Results show that WT and L2-17/CPAF organisms inhibit p65 nuclear translocation at 20 h postinfection (hpi) (Fig. 4A and C). Host cells infected with null organisms showed nuclear translocation (Fig. 4B), which was similar to the result seen in the positive-control TNF-α-treated mock-infected cells (Fig. 4D). The percentage of nuclear translocation in TNF-α-treated mock-infected cells and null-infected cells was >55%, whereas WT, L2-17/CPAF, and mock infections showed less than <15% p65 nuclear translocation (Fig. 4E). IFN-β secretion levels in WT and L2-17/CPAF strains were comparable and significantly lower than those in null-infected cells at 16 and 24 hpi (Fig. 4F).

### CPAF complementation of null mutant strain reduces T3SE protein abundance.

**FIG 5:**
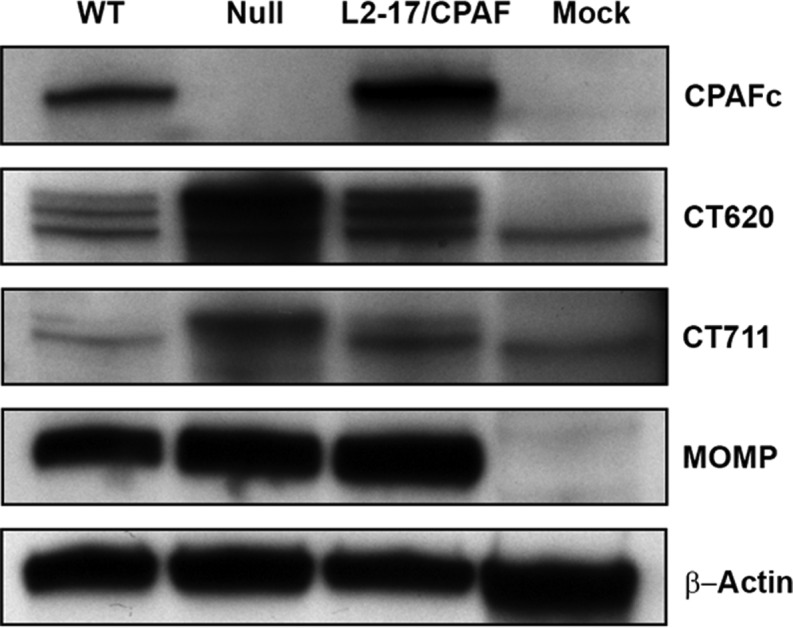
CPAF complementation restores proteolysis of chlamydial DUF T3SEs. Western blot analysis of WT, null, L2-17/CPAF, and mock infections confirms that complementation of CPAF and expression of active CPAFc restore the proteomic reduction of higher-molecular-weight forms of T3SEs CT620 and CT711 in WT and L2-17/CPAF infections. Null-infected cells retain a greater abundance of the higher-molecular-weight form of CT620 and CT711. Host β-actin and chlamydial major outer membrane protein (MOMP) served as loading controls.

Proteomic analysis of WT- and null-infected cells (Fig. 1A and B) revealed that CPAF’s primary chlamydial targets were both structural elements of the T3SS and T3S effector proteins. Western blot analysis of T3SEs in WT, null, L2-17/CPAF, and mock infections showed that the expression of recombinant CPAFc reduces the abundance of higher-molecular-weight fragments of DUF582 effector CT620 and DUF720 effector CT711 (Fig. 5). Coupled with the finding that CPAF does not proteolyze p65 directly, this result indicates that CPAF’s role in virulence is indirect and mediated through either the T3SS or DUF 582/720 T3SE(s).

## DISCUSSION

**FIG 6:**
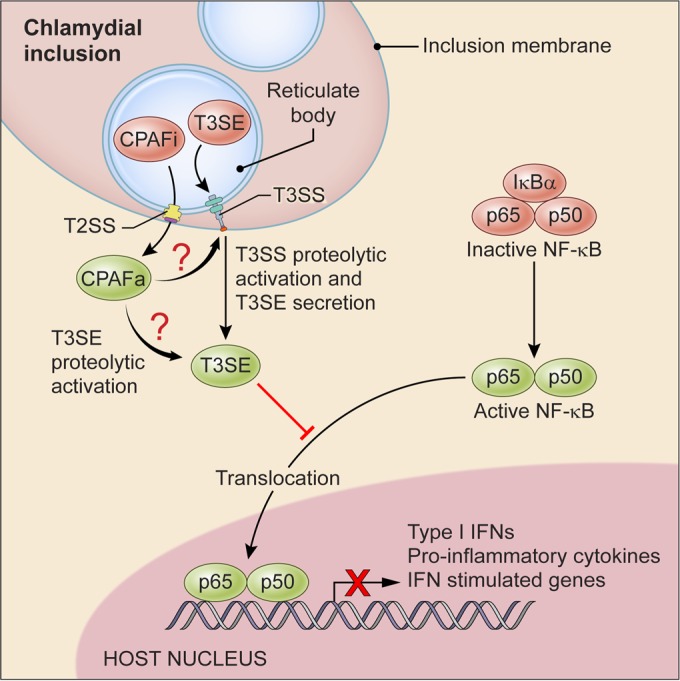
Working model for CPAF and type III secreted proteins in the suppression of host innate immunity. CPAF is produced as a zymogen inactive form (CPAFi) inside the RB and secreted into the host cytoplasm by a type II secretion system (T2SS). Expression of CPAFa inhibits p65 nuclear translocation by mechanisms yet to be defined, resulting in the inhibition of type I interferon and proinflammatory cytokine synthesis. CPAFa proteolysis of critical chlamydial midcycle type III secretion system (T3SS) proteins could function to release type III secreted effectors (T3SEs) from the RB into the host cytoplasm. Alternatively, CPAFa proteolysis of chlamydial T3SEs directly may activate them for virulent function.

The central theme of prior attempts to characterize CPAF’s function was the search for the protease’s substrate(s). Over the past 2 decades, dozens of protein targets were put forth as potential candidates; however, recent reports have shown that insufficient denaturation of CPAF results in nonspecific proteolysis of proteins during sample preparation (15). Consequently, CPAF’s protein target(s) and role in chlamydia virulence remain poorly defined. Recent studies using recombinant CPAF have proposed complement and antimicrobial peptides as *ex vivo* host targets (19, 20); however, CPAF’s *in situ* target(s) remains undefined. In this study, we have taken the appropriate measures to denature CPAF’s potent proteolytic activity prior to proteomic analysis and experimentation (15). Here, we show that CPAF and T3SE proteins work cooperatively as chlamydial virulence factors. We show that this cooperation results in the inhibition of p65 nuclear translocation and suppression of IFN-I and proinflammatory cytokine IL-6 and IL-8 expression. Finally, we propose a working model (Fig. 6) where CPAF activates chlamydial T3S proteins which mediate the suppression of host innate immunity.

Our data clearly identify p65 as a target of CPAF in WT and L2-17/CPAF complemented organism infections; however, p65 shows no evidence of proteolytic cleavage (Fig. 2C), suggesting that CPAF’s role in blocking nuclear translocation is indirect. Our proteomic findings provide strong support for a biological relationship between CPAF and T3S proteins in the pathogenesis of chlamydial infection. Above the 1.5-fold threshold, 18 of the 39 chlamydial proteins found to be less abundant in WT than in null infections were type III-related proteins (see Table S2 in the supplemental material). Many of the proteins identified were T3SEs; however, five proteins were associated with the chlamydial T3SS: CT671, the needle length molecular ruler; CT860 and CT579 translocator components; and CT665 and CT667 class III chaperones, which regulate needle length polymerization (16). The recent report showing that CPAF secretion is mediated by a T2SS (8) would intuitively suggest that any targeting of T3S proteins would happen in the host cytoplasm. Based on our findings, CPAF’s function could be to proteolytically modify midcycle T3SS proteins, thereby activating the secretion of effectors. Interestingly, there is a precedent for this model as proteolytic regulation of injectisomes exists in other Gram-negative pathogens. The *Escherichia coli* protease EspC has been shown to activate injectisomes by cleavage of critical needle-tip and translocation proteins (21). Alternatively, CPAF may directly target chlamydial T3SEs. For example, we show proteolytic processing of CT620 and CT711 in WT-infected cells (Fig. 1B) as previously described (7). How CPAF and T3SE proteins interact is currently unclear; however, the presence of DUF on all of the T3SE proteins identified (7) could be the crucial binding motif necessary for CPAF to interact with the effectors once both are secreted. This is supported by proteolytic processing of DUF582 effector CT620 and DUF720 effector CT711 in both the WT- and L2-17/CPAF complemented organism-infected cells (Fig. 1B and 5). However, we cannot exclude the possibility that chlamydial T3SEs, similarly to other prokaryotic T3SEs, may simply be less stable once secreted into the host cytosol (4).

We similarly cannot exclude the possibility that the *in situ* host proteins identified by proteomics are directly targets of CPAF; however, we favor the explanation where decreased abundance of RIG-I and ISGs (Fig. 2A) and proinflammatory cytokines (Fig. 2I) in WT-infected cells is a direct consequence of blocking p65 nuclear translocation (Fig. 3A), resulting in decreased IFN-β secretion (Fig. 2F and 4F) and thus a reduction in downstream autocrine and paracrine JAK-STAT signaling (Fig. 2D) (22–24). Results showing that, in WT-infected cells, exogenously added rIFN-β can still activate the JAK-STAT pathway (Fig. 2E) support this conclusion. The proteomics data also indicate that the host response is not as robust as that of chlamydiae when comparing WT and null infections (Fig. 1A and 2A). A likely explanation is that the host response is dependent on multiplicity of infection (MOI); thus, a more robust host response would likely be seen if higher MOIs were used.

How might T3SEs block p65 nuclear translocation? They could block p65 translocation by (i) inhibiting IκBα complex formation or degradation, (ii) binding directly to p65, or (iii) blocking nuclear pore function. Precedent for the abovementioned mechanisms exists in viral and bacterial pathogens. *Shigella flexneri* T3SE OspG inhibits degradation of IκBα (25), whereas measles virus V-protein disrupts NF-κB activation by direct binding to p65 (26). The *Salmonella enterica* effector SpvD blocks nuclear pore function, resulting in defective p65 nuclear translocation (27). While numerous pathogenic mechanisms of NF-κB suppression may exist, our proteomic data identify a restricted number of T3SEs as candidate virulence factors (Fig. 1A). The recent development of systems for targeted gene knockout in chlamydiae (28) coupled with monitoring IFN-β secretion by cells infected with T3SE-knockout strains will be an important next step in identifying the specific T3SE(s) and mechanism(s) that inhibit p65 nuclear translocation.

In addition to preventing innate autocrine/paracrine signaling and ISG induction, chlamydial inhibition of IFN-I synthesis presents a logical pathogenic strategy for dampening downstream adaptive T-helper 1 cell (T_H_1) development in humans (29). Our findings would be consistent with the lack of protective immunity in human chlamydial infections, resulting in chronic and recurrent disease (2). Intriguingly, unlike human infections, *Chlamydia muridarum* infections in murine models produce a robust protective immune response after rechallenge (30). This is an apparent dichotomy, as the two strains share CPAF and common T3S effectors; however, T_H_1 development in mice is dependent on IL-12 alone and does not require IFN-I (29). This critical difference in host adaptive immune response might explain the experimental differences between human and mouse infections.

In this study, we have narrowed the focus of CPAF investigation by discovering a novel and restricted function for the cryptic protease. Our *in vitro* findings transform CPAF’s role as a highly nonspecific serine protease, acting primarily on host proteins, to one of specificity and function directed at the inhibition of host innate immunity through the activation of T3S proteins. The recent *in vivo* findings by Yang et al. (18) describe a role for CPAF in promoting the survival of chlamydiae in mouse lower but not upper genital tract tissues. The authors propose that CPAF’s function might be to inhibit the robust innate immunity in the lower genital tract to promote chlamydial survival. Our *in vitro* findings are consistent with this possibility and expand on their observations by providing a mechanism by which CPAF functions to avoid host innate immune defenses.

## MATERIALS AND METHODS

### *Chlamydiae*.

The *Chlamydia trachomatis* L2 RST5 CPAF-sufficient strain and RST17 CPAF-deficient strain were a kind gift from Raphael Valdivia, Duke University (8). The L2-17/CPAF complemented strain was a kind gift of Guangming Zhong, University of Texas San Antonio Medical Center (18). The strains were grown as described in Text S1 in the supplemental material. Elementary bodies were purified as previously described (31).

### Proteomics.

HeLa cells grown in 150-cm^2^ tissue culture (TC) flasks (4 × 10^7^ cells) were infected with WT or null organisms in sucrose-phosphate-glutamic acid medium (SPG), rocked for 1.5 h, and incubated at 37C° in a 5% CO_2_ humidified atmosphere. A multiplicity of infection (MOI) of 5 was used to ensure that monolayers had >95% infection. Cells were harvested by trypsinization at 30 h postinfection (hpi). Trypsinized cells were centrifuged at 500 × *g* for 5 min and resuspended in 1 ml hot 2% SDS-50 mM HEPES (pH 8.2) and boiled for 10 min (15). This method was sufficient to inactive exogenous CPAF activity, as we observed no proteolysis of the host protein vimentin, USF-1, or p65 in lysates by Western blotting (Fig. 2C). Protein extracts from whole-cell lysates were trypsin digested, labeled by isobaric mass tags, and analyzed by shotgun proteomics using nLC-MS/MS on a Q-Exactive Plus mass spectrometer (36) (Thermo Fisher) (see Text S1 in the supplemental material). The nLC-MS/MS data were searched against a human and a *Chlamydia trachomatis* 434Bu database (20,204 and 893 sequences, respectively). Proteomic statistics were performed on experimental groups (WT, null, and mock) done in quadruplicate. If protein hits were not present in 3 or more of the experimental groups, they were discarded. These analyses resulted in a total of 6,903 proteins identified with a 2-peptide 99% confidence threshold (1.0% false-discovery rate [FDR]). Differentially expressed proteins were identified using the Linear Model for Micro Arrays (LIMMA) R package (32). The corresponding *P* values for each comparison were adjusted using the multiple testing procedure developed by Benjamini and Hochberg (33). Protein hits with adjusted *P* values of <0.05 and fold changes of ≥2 were considered statistically significant.

### Western blotting.

HeLa cells were infected in 150-cm^2^ tissue culture (TC) flasks (4 × 10^7^ cells) and processed as described above under “Proteomics.” Samples were standardized, and Western blotting was performed as described in Text S1 in the supplemental material.

### IFN-β, IL-6, and IL-8 enzyme-linked immunosorbent assay (ELISA).

Cell culture supernatants from WT-, null (MOI of 5)-, and mock-infected cells in 24-well plates (4 × 10^5^ cells/well) were collected at 8, 16, and 24 hpi. IFN-β was assayed using the PBL Assay, and IL-6 and IL-8 were assayed using a Bio-Plex Pro Human Cytokine Group I 27-plex panel according to the instructions of the manufacturer.

### IFN-β qRT-PCR.

IFN-β mRNA was harvested from WT-, null (MOI of 5)-, and mock-infected cells in 24-well plates (4 × 10^5^ cells/well) using a Qiagen extraction kit and frozen at −80°C. IFN-β mRNA levels were quantified at 8, 16, and 24 hpi using a 1-step reverse transcription-quantitative PCR (qRT-PCR) method as previously described (34).

### Effect of IFN-β on chlamydial growth.

HeLa cells grown in 24-well plates (4 × 10^5^ cells/well) were pretreated for 24 h with Dulbecco modified Eagle medium 10 (DMEM-10) containing 100 U of recombinant IFN-β (rIFN-β). Medium was removed, and cells were infected (MOI of 1) as described under “Proteomics.” Chlamydial rIFU were isolated from infected cells as previously described (35).

### Immunofluorescence.

HeLa cells grown on coverslips (4 × 10^5^ cells/coverslip) were WT, null (MOI of 5), and mock infected. Infected cells were fixed at 20 hpi with 4% formaldehyde in phosphate-buffered saline (PBS) for 30 min, blocked for 1 h with PBS containing 0.3% Triton and 100 mg/ml goat serum, and incubated with rabbit or mouse anti-CT620, anti-p65, anti-HSP60, or anti-CPAF. Coverslips were washed, incubated with DAPI or Alexa Fluor 488 or 555 secondary antibodies, mounted using ProLong Gold, and imaged by confocal microscopy. Cells treated with 150 ng/ml recombinant human TNF-α were similarly fixed and stained with anti-p65 and served as a positive control for p65 nuclear translocation in WT- and null-infected cells. Images were collected with a 63× 1.4-numerical-aperture (NA) oil objective on a Zeiss LSM 880 laser scanning microscope with an Airyscan detector. Z-stacks were collected at 0.2-µm intervals. All images were processed in Zen Blue and Zen Black (Carl Zeiss Imaging).

## SUPPLEMENTAL MATERIAL

Table S1  Host and chlamydial proteome spreadsheet for WT- versus null mutant- versus mock-infected cells. The table includes all proteins identified in WT, null, and mock infections with adjusted *P* values and fold changes included. Table S1, XLSX file, 0.4 MB

Table S2  Chlamydial proteins exhibiting a >1.5-fold difference between WT- and null-infected cells. Table S2 includes all chlamydial proteins identified above the 1.5-fold cutoff for WT- versus null-infected cells. Table S2, XLSX file, 0.02 MB

Text S1  Supplemental experimental procedures. Download Text S1, DOCX file, 0.02 MB
