# Supplementary material for: Chlamydial Protease-Like Activity Factor and Type III Secreted Effectors Cooperate in Inhibition of p65 Nuclear Translocation
Source: mBio. 2016 Sep 27;7(5):e01427-16. doi: 10.1128/mBio.01427-16 (PMC5040114; doi:10.1128/mBio.01427-16)
Supplement: Text S1 — Supplemental experimental procedures. Download [file mbo005163007s1.docx]

**Supplemental Experimental Procedures**

**Chlamydiae**

*Chlamydia trachomatis* parental L2 RST5 wild-type and RST17 CPAF null mutant strains were a kind gift Dr. Raphael Valdivia, Duke University. The strains were grown in HeLa cells using high-glucose-containing Dulbecco's modified Eagle medium supplemented with 10% fetal bovine serum (DMEM-10), 2 mM L-glutamine, 1 mM HEPES, 1 mM sodium pyruvate, 0.055 mM beta-mercaptoethanol and 10 µg/ml Gentamicin at 37°C with 5% CO_2._  Elementary bodies were purified as previously described (1).

**Nanoflow Liquid Chromatography-Tandem Mass Spectrometry Sample Preparation**

WT, null and mock infected cells samples were prepared as described in Proteomics (see Experimental Procedures). Protein concentration of the samples, in the presence of SDS, was determined using a Pierce Bicinchoninic Acid (BCA) Protein Assay kit (Thermo Fisher Scientific), using bovine serum albumin (BSA) in the same concentration of SDS as the reference standard. Samples (100 µg each in volume top-up by water, LC/MS Optima water, Fisher Scientific, to 100 µl) were reduced by adding dithiothreitol (DTT, Sigma) to a final DTT concentration of 100 mM, and heating at 80°C for 10 min. After the samples were cooled back to room temperate, an equal volume of urea exchange buffer (UEB, 8 M urea, GE Healthcare, 50 mM HEPES, pH 8) was added and incubated for 10 minutes at room temperature. Nanosep 10k Omega centrifugal filters (Pall Life Sciences) were prepared by centrifugation at 10,000xg of 200 µl of water, followed by 200 µl of UEB, centrifuging each time to near dryness (~10 µl retentate). Each sample was then transferred to a prepared centrifugal filter and filtered at 10,000xg, followed by three additional washes, with 250 µl of UEB per wash, without centrifuging the filter to dryness. Following the UEB washes, samples were alkylated using 100 µl of 50 mM iodoacetamide (IAA, Sigma), in UEB added to the samples on the filter, mixed for 1 min at 600 RPM on a shaker at room temperature, and then incubated for 20 minutes at room temperature in the dark. Samples were centrifuged, followed by three washes with 100 µl of UEB, and then washed twice with 150 µl 50 mM HEPES, pH 8. To remove DNA, 50 µl of a benzonase solution (50 mM magnesium chloride, Invitrogen, 50 mM HEPES pH 8, 100 U benzonase, EMD) was added to each sample on filter, mixed at 600 RPM for 2 minutes, and incubated for 30 minutes at room temperature. Samples were then centrifuged as described above with three washes, using 100 µl of 50 mM HEPES, pH 8, per wash. The centrifugal filter cartridges were then transferred to a fresh collection tube, and 50 µl of HEPES pH 8 containing 1.5 µg of trypsin (Pierce Trypsin Protease, MS-Grade, Thermo Fisher Scientific) was added to the samples. The samples were mixed for 1 minute at 600 RPM, and incubated at 37°C overnight in a humidified chamber. After digestion, the tryptic peptides were collected, and the filters were washed twice with 50 µl of 50 mM HEPES, pH 8, with the washes collected and pooled for each sample. The tryptic peptide samples were concentrated to near-dryness (1-5 µl) using a vacuum centrifuge (Savant Speed/Vac Concentrator, Thermo Fisher Scientific). The samples were then brought to a total volume of 59 µl with 50 mM HEPES pH 8 and peptides were labelled using the 6-plex TMT kit (Thermo Fisher Scientific) according to the manufacturer’s instructions. Briefly, the TMT Label Reagents were equilibrated to room temperature and then resuspended in 41 µl of acetonitrile (Fisher Scientific), and incubated for 5 minutes at room temperature with occasional mixing. The trypsin-digested samples were then added to the TMT Label Reagent, and incubated overnight at room temperature. The TMT labeling reaction was quenched by adding 8 µl of 5% hydroxylamine (Thermo Fisher) in 50 mM HEPES pH 8 and incubation for 15 minutes at room temperature. The samples were then concentrated to near-dryness under vacuum centrifugation. The trypsin-digested, TMT-labelled samples were brought up to a final volume of 35 µl with water. To ensure one-to-one mixing of all samples within a 6-plex TMT experiment, equal volumes (2 µl) of each labelled sample were mixed with nano-LC buffer A (2% acetonitrile, 0.1% formic acid) to a final volume of 60 µl and analyzed by nano-LC/MS/MS using a 2-hour gradient (see below). Histograms of count versus ratio for each TMT report ion pair were generated using the peak list from Proteome Discoverer (v2.0, Thermo Fisher Scientific) and an in-house Perl script. The median ratio (histogram centroid) for each report ion pair was used to make minor corrections to the final 6-plex mixing volumes, with the final mixing volumes calculated to yield unity median TMT ratios, for a total volume 40 µl of the TMT 6-plex mix.

**Peptide Fractionation by Off-line, High pH Reversed-Phase Liquid Chromatography**

The TMT 6-plex mixed samples were spiked with 4 µl of 200 mM ammonium formate (Fisher Scientific, EMD), pH 10, to match LC buffer A (20 mM ammonium formate, pH 10). Sample (40 µl) was loaded onto a Waters XBridge C18 guard column (10 mm long, 2.1 mm inner diameter, 3.5 µm particles) on a micro-flow 1200 Series HPLC pump (Agilent Technologies). Peptides were then separated on a Waters XBridge C_18_ analytical column (100 mm long, 2.1 mm inner diameter, 3.5 µm particles) using a 80-minute gradient of 3-75% LC buffer B (20 mM ammonium formate, 90% acetonitrile, pH 10) at a constant flow rate of 150 µl/min. A total of 12 fractions were collected across the peptide elution profile. These fractions were concentrated to near-dryness under vacuum centrifugation and resuspended in 40 µl of nano-LC buffer A (2% acetonitrile, 0.1% formic acid).

**Nano-LC/MS/MS**

Each off-line LC fraction was sequentially analyzed using a nano-flow Easy 1000 in-line to a Q-Exactive Plus mass spectrometer with a nano-electrospray ion source at 2.0 kV (Thermo Fisher Scientific). The peptide fractions were loaded (2 µl) and washed (10 µl at 750 bar) on a C_18_-reversed phase Easy-Spray column (Thermo Fisher Scientific, 50 cm long, 75 µm inner diameter, 2.0 µm particles) with 100% nano-LC buffer A. Peptides were eluted using a 120-min linear gradient of 2-30% nano-LC buffer B (98% acetonitrile, 0.1% formic acid) at a constant flow rate of 200 nl/min. Total nano-LC/MS/MS run-time was 160 minutes, including the loading, linear gradient, 15-minute column wash at 95% buffer B, and the column re-equilibration (5 µl nano-LC buffer A). The full-MS survey scans were acquired in the Orbitrap over *m/z* 300-1700 with a target resolution of 70,000 at *m/z* 200, AGC target of 3e6, and maximum injection time of 80 ms. Data-dependent acquisition method was used, dynamically choosing the top 15 abundant precursor ions from each survey scan with an isolation width of *m/z* 3 for fragmentation by HCD (34% normalized collision energy). The intensity threshold for selecting a precursor ion for fragmentation was 1e5 ions, with charge state recognition of 2-5, and a dynamic exclusion for 10 s. The fragment ion MS2 scans were also acquired in the Orbitrap over a dynamic *m/z* range with a target resolution of 17,500 at *m/z* 200, AGC target of 2e5, and maximum injection time of 100 ms. Lock Mass was used with polysiloxane.

**Data Processing**

All spectra were processed using Proteome Discoverer (v2.0, Thermo Fisher Scientific) and database searching was done with Mascot v2.5 (Matrix Science). Searches were performed against the SwissProt database (2015_05) restricted to human databases (20,204 sequences) and a *Chlamydia trachomatis* 434Bu database (893 sequences) from NCBI’s Genome database of bacteria. The decoy database option was selected, and the following search parameters were used: Carbamidomethyl (C) was selected as a fixed modification, Oxidation (M) as a variable modification, fragment ion mass tolerance of 0.5 Da, parent ion tolerance of 10 ppm, and trypsin enzyme with up to 1 missed cleavage. TMT 6plex was selected as the Quantitation method. Mascot search results were imported into Scaffold Q+ v4.4 (Proteome Software) and filtered using 0.1% FDR for peptides, 1.0% FDR for proteins, and at least 2 peptides per protein.

**Proteomic Statistics**

Proteomic statistics was performed on experimental groups (WT, null and mock) done in quadruplicate. If protein hits were not present in 3 or more of the experimental groups they were discarded. These analysis resulted in a total 6,903 proteins identified with a 2-peptide, 99% confidence threshold. Protein hits were analyzed for statistical significance as previously described (2). A linear regression model was fitted to identify differentially expressed proteins (3). A Benjamini-Hochberg correction procedure (4) was performed on the p-values and only those proteins with corrected p-values <0.05 were considered to be statistically significant.

**Western Blotting**

WT, null and mock infections in t150 flasks were performed as described in Infection Protocol and lysates processed for proteomics as described in Proteomics. Protein concentration was determined by the BCA assay (Pierce) and then diluted into Laemmli sample buffer (50mM Tris-HCL [pH 6.8], 10% glycerol, 2% SDS, 1% 2-mercapthoethanol, and 0.1% bromophenol blue). Samples of equivalent protein load were resolved at 60V for 3 hours in 4-20% SDS-PAGE gradient gels (Biorad #345-0032). Proteins were transferred onto PVDF membranes and 0.2um polyvinyl difluoride (PVDF) membranes at 1 Amp for 30 minutes with the Semi-dry Apparatus (Biorad). Membranes were subsequently blocked with 5% (w/v) skim milk and Tris buffered saline with Tween-20 (TBST; 50 mM Tris, 150mM NaCl, 0.05% Tween 20, pH 7.4) and probed with a variety primary antibodies. Human primary antibodies were: α-IFIT3 (Gift of Dr. Luna Zaritsky), α-NF-kB p65 (Cell Signaling D14E12) 1:1000, α-MX2 (Abcam 67388) 1:1000, α-STAT2 (Millipore, #07140) 1:1000, α-pSTAT2-Tyr690 (Millipore, #07224) 1:1000, α-STAT1 (Cell Signaling #9172S) 1:1000, α-pSTAT1-Tyr701 BD (Biosciences, #612132) 1:1000, α-OAS2 (Santa Cruz #271117) 1:800, α-Beta Actin (Cell Signaling #4970S) 1:3000. Chlamydial primary antibodies were: α-CT620 1:800, α-CT621 1:800, α-CT711 1:800 (Gift of Dr. Agathe Subtil), α-Chlamydial Protease-like Activation Factor 1:10 (Gift of Dr. Guangming Zhong), α-Heat Shock Protein 60 1:200 (Caldwell). Appropriate secondary horseradish peroxidase or alkaline phosphate conjugated anti-mouse, anti-rabbit, anti-goat antibodies from Western Breeze kits were used to detect immune complexes (Life Technologies). Protein bands were developed visualized on Amersham Hyperfilm (Genesee Scientific #83-618) and imaged with a TI-BA Series 2000A Film Processor instrument.

**IFN-β ELISA and Cytokine Analysis**

Confluent HeLa cell monolayers in 24-well plates were infected as described in Infection Protocol. Cell culture supernatants from WT, null and mock infected cells were collected at 8, 16, 24hpi. IFN-β, IL-6 and IL-8 concentrations were measured per the manufacturer’s instructions PBL Assay Science (#41410-1) and Bio-Plex Pro Human Cytokine Group I 27-plex panel (#M50-0KCAF0Y).

**IFN-β qRT-PCR**

Infected cells were washed with 1X PBS and IFN-β mRNA was harvested using a RNeasy Mini kit (Qiagen #74104) with RNase-Free DNase Set (Qiagen #79254) and frozen at -80°C. IFN-β mRNA levels were quantified at 8, 16, 24 hpi using a 1-step qRT-PCR method as previously described (5).

**Effect of IFN-β on Chlamydial growth**

HeLa cells were pretreated for 24 hours with DMEM-10 containing 100U of human rIFN-β (R&D Systems #8499-IF-010). Media was removed and cells were infected using a MOI of 1. Chlamydial rIFUs were isolated from infected cells was determined as previously described (6).

**Immunofluorescence**

Confluent HeLa cell monolayers on coverslips in 24-well plates were infected as described in the Infection Protocol. A portion of the mock infected coverslips were either untreated or treated with 150ng/ml recombinant human TNF-α (hTNF-α) (Cell Signaling #8902) for 1 hour at 37C° to serve as negative and positive controls for p65 nuclear translocation. At 20hpi, cells were fixed in 4% formaldehyde/1X PBS solution for 30 minutes at room temperature, blocked for 1 hour in a 1X PBS solution containing 0.3% Triton and 100mg/ml goat serum at room temperature and stained with 1X PBS, 1% BSA, 0.3% Triton solution containing various antibodies. Primary antibodies: α-CT620 1:800 (Gift of Dr. Subtil), α-p65 at 1:800 (Cell Signaling #D14E12), α-hsp60 1:400 (Caldwell), α-CPAF (Gift of Dr. Zhong). Secondary antibodies: 1:400 (Alexa-Fluor 555 goat anti-mouse #A21424, Alexa Fluor 488 goat anti-rabbit #A11034). Coverslips were stained with DAPI at 1:1000 in AB for 10 minutes and mounted using Prolong Gold. High resolution images were captured using a Zeiss 880 laser scanning microscope with Airyscan detector. Z-stack projections were imaged at an interval of 0.2um. All images were processed in Zen Blue and Zen Black (Carl Zeiss Imaging).

# **References**

1. **Caldwell, HD, Kromhout, J & Schachter, J.** 1981. Purification and partial characterization of the major outer membrane protein of Chlamydia trachomatis. *Infection and Immunity,* **31**:1161-1176.
2. **Simon, PF, McCorrister, S, Pingzhao, H, Chong, P, Silaghi, A, Westmacott, G, Coombs, KM & Kobasa, D.** 2015. Highly Pathogenic H5N1 and Novel H7N9 Influenza A Viruses Induce More Profound Proteomic Host Responses than Seasonal and Pandemic H1N1 Strains. *Journal of Proteome Research,* **14**:4511–4523.
3. **Smyth, GK.** 2004. Linear models and empirical Bayes methods for assessing differential expression in microarray experiments. *Statistical Applications in Genetics and Molecular Biology,* **3**(1).
4. **Benjamini, Y & Hochberg, Y.** 1995. Controlling the false discovery rate: a practical and powerful approach to multiple testing. *Journal of the Royal Statistical Society,* B57(1), 289–300.
5. **Zaritsky, LA, Besaul, JR & Zoon, KC**. 2015. Virus Multiplicity of Infection Affects Type I Interferon Subtype Induction Profiles and Interferon-Stimulated Genes.. *Journal of Virology,* **89**:11534-48.
6. **Morrison, RP, Feilzer, K & Tumas, DB.** 1995. Gene knockout mice establish a primary protective role for major histocompatibility complex class II-restricted responses in Chlamydia trachomatis genital tract infection. *Infection and Immunity,* **63**:4661-4668.
